# Cutaneous Changes Beyond Psoriasis: The Impact of Biologic Therapies on Angiomas and Solar Lentigines

**DOI:** 10.3390/medicina61040565

**Published:** 2025-03-22

**Authors:** Florin Ciprian Bujoreanu, Diana Sabina Radaschin, Ana Fulga, Laura Bujoreanu Bezman, Carmen Tiutiuca, Mihaela Crăescu, Carmen Pantiș, Elena Niculet, Alina Pleșea Condratovici, Alin Laurențiu Tatu

**Affiliations:** 1 Department of Dermatology, “Saint Parascheva” Infectious Disease Clinical Hospital, 800179 Galati, Romania; florin.bujoreanu@gmail.com (F.C.B.); dianaradaschin@yahoo.com (D.S.R.); alin.tatu@ugal.ro (A.L.T.); 2 Faculty of Medicine and Pharmacy, “Dunarea de Jos” University of Galati, 800385 Galati, Romania; ana.fulga@ugal.ro (A.F.); mihaela.craescu@ugal.ro (M.C.); helena_badiu@yahoo.com (E.N.); alina.plesea@ugal.ro (A.P.C.); 3 Multidisciplinary Integrated Centre of Dermatological Interface Research (MICDIR), “Dunarea de Jos” University of Galati, 800385 Galati, Romania; 4 Department of Surgical Disciplines, Faculty of Medicine and Pharmacy, University of Oradea, 1 University Street, 410087 Oradea, Romania; pantisc@yahoo.com

**Keywords:** psoriasis, biologic therapy, cytokines, angiomas, solar lentigos, digital dermoscopy, reflective confocal microscopy

## Abstract

*Background and Objectives*: Psoriasis is a chronic inflammatory skin disease, and biologic therapies have revolutionized treatment by targeting key cytokine pathways. While these therapies effectively control psoriatic lesions, their impact on other cutaneous structures, such as cherry angiomas and solar lentigines, remains unclear. Angiomas are benign vascular proliferations influenced by systemic inflammation and hormonal factors, whereas solar lentigines are UV-induced pigmentary lesions associated with aging and sun exposure. This study aimed to assess the impact of biologic therapies on the development of these lesions in psoriasis patients. *Materials and Methods*: This retrospective observational study was conducted over a five-year period (2019–2024) at a tertiary dermatological center in Southeastern Europe. Clinical and demographic data, including treatment history, were extracted from medical records, while digital dermoscopy was used to assess lesion progression. Statistical analyses evaluated associations among biologic therapy classes, systemic inflammation, and cutaneous lesion development. *Results*: Angioma prevalence was significantly higher among postmenopausal women and those with osteoporosis, suggesting a hormonal influence on vascular proliferation. Patients with psoriatic arthritis had a greater angioma burden, reinforcing the role of chronic inflammation in angiogenesis. IL-23 inhibitors were linked to increased angioma formation compared to TNF-α inhibitors, while methotrexate and UVB therapy appeared to have a protective effect. Solar lentigines were more frequent in postmenopausal women and in patients with systemic inflammatory conditions. In contrast, smoking and moderate alcohol consumption were associated with lower lesion counts. *Conclusions*: Our findings suggest that biologic therapies, particularly IL-23 inhibitors, may contribute to angiogenesis and pigmentary changes in psoriasis patients, highlighting the influence of systemic inflammation on vascular and melanocytic activity. Additionally, TNF-α inhibitors and NSAIDs were associated with an increased prevalence of solar lentigines, while methotrexate and UVB therapy appeared to have a protective effect. Given these associations, further research is needed to elucidate the underlying mechanisms and refine treatment strategies to optimize dermatologic care for psoriasis patients.

## 1. Introduction

Plaque psoriasis, or psoriasis vulgaris, is the most prevalent form of psoriasis, affecting approximately 80–90% of individuals diagnosed with the condition. This chronic inflammatory cutaneous disease manifests clinically as erythematous plaques covered with silvery scales, primarily impacting the skin and joints, and is associated with various comorbidities [1]. The management of plaque psoriasis has evolved significantly with the advent of systemic treatments and biologic therapies, reflecting a deeper understanding of the disease’s pathophysiology [2]. The clinical assessment of plaque psoriasis severity often utilizes the Psoriasis Area and Severity Index (PASI) score, which evaluates parameters such as erythema, induration, and desquamation, along with the percentage of body surface area affected.

Psoriasis is a chronic inflammatory skin condition that significantly impacts the quality of life (QoL) of affected individuals. The Dermatology Life Quality Index (DLQI) is commonly utilized to assess the extent of this impact, revealing that a substantial proportion of patients experience moderate to severe impairment in various aspects of their lives, including emotional, social, and occupational components. A study conducted in China indicated that over 84% of psoriasis patients reported varying degrees of QoL impairment, which was closely associated with disease severity and the psychological burden arising from the condition, such as anxiety and depression [3].

The psychosocial implications of psoriasis are profound, as the disease is often accompanied by stigma and social isolation, which further exacerbate the emotional toll on patients [4]. Moreover, the visibility of psoriatic lesions, particularly in areas such as the face and hands, can lead to increased self-consciousness and social withdrawal, compounding the negative effects on mental health.

Systemic treatments for plaque psoriasis encompass a range of therapeutic options, including traditional immunosuppressants like methotrexate and cyclosporine, as well as novel biologic therapies that target specific immune pathways [5].

Methotrexate (MTX) is a well-established systemic therapy for psoriasis vulgaris, widely used for its ability to target both keratinocyte hyperproliferation and the chronic inflammatory processes underlying the disease. Given its cost-effectiveness and well-documented clinical benefits, methotrexate remains an important first-line option in the systemic management of psoriasis [6]. Despite its therapeutic benefits, the use of methotrexate is not without risks. The drug is associated with hepatotoxicity and immunosuppressive effects, which necessitate careful monitoring and management [7]. Methotrexate’s immunosuppressive effects may increase susceptibility to opportunistic infections, particularly with long-term use. This risk necessitates careful evaluation of its therapeutic benefits alongside potential adverse effects when considering methotrexate for psoriasis management [8].

Biologic therapies have revolutionized the treatment of moderate to severe plaque psoriasis, offering long-term disease control and improving patients’ quality of life. These therapies, which target specific pathways involved in the pathogenesis of psoriasis, have been shown to achieve substantial improvements in skin clearance, with many patients reaching PASI 90 or even complete skin clearance (PASI 100) [9].

Biologics, such as tumor necrosis factor (TNF) inhibitors and interleukin (IL) inhibitors, have revolutionized the management of plaque psoriasis by providing a targeted therapeutic approach that addresses the underlying immunological dysfunction. The introduction of biologic therapies has not only improved psoriasis treatment but has also had a profound impact on patients’ daily lives and overall well-being. Biologic therapies targeting TNF-α, IL-17, and IL-23 effectively control psoriasis by reducing chronic inflammation and improving skin clearance. In addition to alleviating physical symptoms, they contribute to an enhanced quality of life by restoring patient confidence, increasing energy levels, and supporting overall well-being and daily functioning [10].

Solar lentigos are benign hyperpigmented lesions that develop primarily in areas chronically exposed to ultraviolet (UV) radiation. These lesions appear as small, flat, brown macules, most commonly affecting elderly adults, as they reflect cumulative sun exposure and photodamage over time [11]. Although solar lentigos most frequently occur on the dorsal hands, they are also commonly found on the face, back, shoulders, arms, and feet, particularly in individuals with lighter skin types who have experienced prolonged sun exposure. Their appearance is often uniform in color with well-defined borders, distinguishing them from other pigmentary disorders such as melasma or ephelides [12]. However, differentiating solar lentigos from premalignant or malignant lesions, such as lentigo maligna, remains essential, particularly in individuals with significant sun exposure or a history of skin cancer [11].

Their pathogenesis is largely driven by prolonged UV exposure, which stimulates melanocyte proliferation and melanin overproduction, leading to persistent pigmentation. In addition to UV radiation, factors such as chronic inflammation and oxidative stress play a role in melanocyte activation, influencing pigmentary alterations associated with aging and photoaging. Histologically, solar lentigos exhibit epidermal hyperplasia, increased melanin deposition in basal keratinocytes, and an expanded rete ridge pattern, which distinguishes them from other hyperpigmented lesions. The role of dermal fibroblasts in lentigo formation has also been increasingly recognized, as these cells contribute to melanocyte regulation through paracrine signaling, further influencing pigmentation changes over time [13].

Several treatment modalities are available for solar lentigos, particularly for individuals seeking cosmetic improvement. Among these, laser-based therapies are widely used due to their ability to target melanin selectively. Various lasers emitting specific wavelengths have been shown to effectively lighten solar lentigos by breaking down excess pigment, allowing the body’s immune system to clear the pigmented cells [14].

Despite the availability of effective treatments, prevention remains the most effective strategy for managing solar lentigos. The consistent use of broad-spectrum sunscreens, protective clothing, and UV avoidance measures significantly reduces their development and progression. Given their strong association with chronic sun exposure, solar lentigos serve as biological markers of cumulative photodamage, reinforcing the importance of photoprotection in skin aging and skin cancer prevention [11].

Angiomas are common benign vascular lesions characterized by small red or purple papules formed by dilated capillaries. These lesions are particularly frequent in adults and can increase in number with age, hormonal changes, and certain medical conditions [15]. These benign tumors are characterized by an abnormal proliferation of blood vessels and can manifest in various forms, including cherry angiomas, cavernous angiomas, and lymphangiomas, and are often classified based on their location and histological features [16]. Cherry angiomas, also known as senile angiomas, are small, red lesions commonly found on the skin, particularly in older adults, while cavernous angiomas, also known as cavernous hemangiomas, are more complex vascular malformations that can occur in the central nervous system [15]. They are typically harmless and do not require treatment unless they become bothersome or are cosmetically undesirable [17]. The prevalence of cherry angiomas increases with age, and they are thought to be associated with genetic factors and environmental influences [18]. Recent studies have also explored the genetic and environmental factors associated with the development of angiomas. For instance, cherry angiomas have been linked to genetic mutations and may serve as markers for skin damage in certain populations [19]. Furthermore, the prevalence of angiomas can vary with age and gender, with men often exhibiting a higher incidence of senile angiomas compared to women [20].

While biologic therapies have significantly improved psoriasis management, their broader impact on other skin structures remains unclear. Clinical observations suggest a possible link between these treatments and secondary skin changes, such as angiomas and solar lentigos, yet this area has not been systematically explored. Given the chronic inflammatory nature of psoriasis and the immune-modulating effects of biologic therapies, it is possible that these factors could contribute to vascular and pigmentary alterations. This study aims to investigate these potential associations, and by addressing this gap, we hope to contribute to a better understanding of how biologic therapies may affect the skin beyond psoriatic lesions, while also emphasizing the need for further research in this area.

## 2. Materials and Methods

Design: The current study was conducted as a retrospective observational study, which was carried out in the Dermatology Department of ”Sf. Parascheva” Clinical Infectious Diseases Hospital in Galati, Romania. It included 60 patients with moderate to severe forms of psoriasis vulgaris who were undergoing biological therapies over a 5-year period (2019–2024).

Clinical, demographic, and treatment-related data were extracted from medical records, while the photographing and documentation of cutaneous lesions in study participants were conducted using digital dermoscopy. The extent of the cutaneous lesions and their evolution during treatment were assessed using the Psoriasis Area and Severity Index (PASI) and the Dermatology Life Quality Index (DLQI).

Inclusion Criteria:

The inclusion criteria included adults with a confirmed histopathological diagnosis of plaque psoriasis, who are undergoing biological therapies from all available therapeutic classes (TNF-α inhibitors, IL-17 inhibitors, IL-23 inhibitors) and have a complete medical history available.

Exclusion Criteria:

The exclusion criteria for this study included patients with insufficient medical records, those who discontinued biologic therapy, and individuals who were initially eligible but no longer met the national protocol criteria for biologic treatment. Additionally, patients with absolute contraindications to biologic therapy, those receiving other systemic therapies apart from biologics, and individuals with milder forms of psoriasis were also excluded.

Data collection and variables:

Patient data were retrieved from electronic and physical medical records, as well as from dermoscopic imaging databases, and the following variables were analyzed:

Demographics and Lifestyle Factors: age, sex, BMI, nutritional status, environmental background, smoking and alcohol consumption history, chronic UV exposure history, and phototype classification.

Psoriasis characteristics and severity: clinical manifestations, including psoriatic arthropathy and its distribution (axial/peripheral), as well as genital, palmoplantar, and nail involvement. Psoriasis severity was assessed using Psoriasis Area and Severity Index (PASI) scores at baseline, and at 3, 6, and 12 months, along with Dermatology Life Quality Index (DLQI) scores recorded throughout the follow-up period.

Biologic therapy and other treatments: This study included an analysis of current and past biologic therapies, dividing patients into groups based on their exposure to TNF-α inhibitors, IL-23 inhibitors, and IL-17 inhibitors. Additionally, prior use of methotrexate, PUVA therapy, UVB therapy, NSAIDs, chemoprophylaxis with isoniazid for tuberculosis prevention, and beta-blockers was documented.

Comorbidities: The presence of relevant systemic comorbidities was documented, including menopausal status, osteoporosis, cardiovascular diseases (hypertension, ischemic heart disease), hepatic, respiratory, and renal diseases, endocrine disorders (diabetes, thyroid dysfunction), and a history of surgical procedures. Other factors assessed included a history of sunburn and the presence of bacterial, viral, or fungal skin infections.

Dermoscopy and skin lesion examination: The total number of angiomas and solar lentigos, including both pre-existing and newly developed lesions, was recorded. Additionally, other dermatologic findings, including total nevi count, actinic keratoses, seborrheic keratoses, keratoacanthoma, skin tags, and non-melanoma skin cancers (NMSC), were assessed through dermoscopic and clinical evaluations.

Statistical analysis: The selected patient data were entered into an Excel table, then imported and processed with the help of SPSS 29.0. The data were sorted into categories, and then the frequency distribution was carried out. Possible influences between the data were investigated with the help of the Fisher’s and chi-squared tests. Descriptive statistics were applied to the quantitative variables, and possible differences were investigated using parametric and non-parametric tests. The following tests were used for sample comparison: the Kolmogorov–Smirnov test, the *t*-Student test, the ANOVA test, the Mann–Whitney test, and the Kruskal–Wallis test. To investigate the association between two quantitative variables (e.g., the influence of age, duration of biologic therapy, disease duration, and initial PASI and DLQI scores on the number of melanocytic nevi initially captured, the number of angiomas, lentigos, and other cutaneous structures), we calculated Pearson’s correlation coefficient (r), together with its significance level and the corresponding 95% confidence interval, which allowed us to characterize the direction and intensity of this association. Statistically significant values were defined as *p* < 0.05, while values of *p* < 0.01 were considered highly statistically significant.

Ethical considerations: This study was conducted in accordance with the Declaration of Helsinki and received ethical approval from the Ethical Committee of “Saint Parascheva” Clinical Hospital of Infectious Diseases, Galati, Romania (Approval No. 2/4, dated 26 March 2024), the Ethical Committee of the Medical College of Galati (Approval No. 160, dated 15 February 2024), and the University Ethics Committee of “Dunărea de Jos” University of Galati (Approval No. 14, dated 30 May 2024).

All patients included in this study have given their informed consent. All patient data were fully anonymized to ensure confidentiality.

## 3. Results

The mean duration of biologic therapy in the study cohort was 62.38 ± 6.01 months, with a range spanning from 10 to 180 months (Table 1).

Three types of biologic agents were administered: IL-17 inhibitors, which were the most frequently prescribed (41.7% of patients), followed by TNF-α inhibitors (30.0%) and IL-23 inhibitors (28.3%) (Table 2).

The patients’ disease duration varied between 4 and 46 years, with an average of 24.62 ± 1.56 years (Table 3).

At baseline, the mean PASI score was 26.24 ± 1.37, significantly decreasing by 51.6% to 12.71 ± 0.98 at three months, 77.8% to 5.81 ± 0.57 at six months, and 91.3% to 2.28 ± 0.31 at 12 months (*p* < 0.001 for all comparisons). A parallel decline in DLQI scores was documented, with a decrease from 21.07 ± 0.81 at baseline to 2.00 ± 0.39 at 12 months, signifying a 90.5% improvement in terms of quality of life (Table 4).

These observations suggest a considerable and stable response to biologic therapy, with a substantial proportion of patients reaching a PASI 90 score or nearly complete disease clearance by the one-year point. The observed improvements align with those reported in clinical trials, reconfirming the real-world efficacy of biologics.

### 3.1. Angioma Distribution in Patients Receiving Biologic Therapy

Women exhibited significantly higher angioma counts than men (11.90 ± 6.18 vs. 3.33 ± 2.12; *p* < 0.05), while urban patients showed a slightly greater angioma burden compared to those in rural areas (6.45 ± 5.97 vs. 5.56 ± 4.91). Interestingly, smokers and high alcohol consumers had fewer angiomas than non-smokers (4.04 ± 3.60 vs. 7.82 ± 6.39; *p* < 0.05) and non-drinkers (4.25 ± 4.01 vs. 9.08 ± 6.54; *p* < 0.05) (Table 5).

Postmenopausal women (13.44 ± 5.79) and patients with osteoporosis (10.88 ± 4.79) had markedly higher angioma counts than premenopausal women (5.75 ± 3.40; *p* < 0.01) and those without osteoporosis (5.46 ± 5.46; *p* < 0.01) (Table 6).

Patients not receiving biologic therapy (biologic-naïve: 5.89 ± 5.55) and those treated with TNF-α inhibitors (5.06 ± 4.39) had lower angioma counts than those on IL-23 inhibitors (6.94 ± 7.00). Additionally, individuals using beta-blockers had significantly higher angioma counts compared to non-users (9.93 ± 5.95 vs. 5.04 ± 5.09; *p* < 0.001).

Patients with psoriatic arthritis had increased angioma counts (9.64 ± 6.60 vs. 3.71 ± 3.09; *p* < 0.001). Notably, those with axial and peripheral joint involvement exhibited markedly higher angioma counts (>10.00) compared to those without joint disease (3.76 ± 3.12; *p* < 0.001) (Table 7).

Patients with endocrine dysfunction had the highest angioma counts (17.00 ± 7.21 vs. 5.61 ± 5.02; *p* < 0.001), while individuals with cardiovascular disease (8.68 ± 6.06 vs. 4.74 ± 4.91; *p* < 0.01) and hypertension (8.28 ± 6.01 vs. 4.69 ± 4.93; *p* < 0.01) also had elevated angioma numbers. Similarly, patients with musculoskeletal disorders showed an increased angioma burden (8.21 ± 5.01 vs. 5.57 ± 5.74; *p* < 0.05) (Table 8).

PUVA therapy (11.86 ± 7.77 vs. 5.43 ± 4.93; *p* < 0.01) and chemoprophylaxis with isoniazid (7.41 ± 6.18 vs. 4.22 ± 4.07; *p* < 0.05) were associated with significantly higher angioma counts. In contrast, patients who underwent methotrexate or UVB therapy had lower angioma counts, suggesting a potential protective effect (Table 9).

### 3.2. Solar Lentigo Distribution in Patients Receiving Biologic Therapy

Women (17.85 ± 15.17) and rural patients (16.72 ± 11.24) had slightly higher solar lentigo counts (Table 10).

Smoking also appeared to influence lentigo formation, with smokers showing significantly lower counts than non-smokers (10.96 ± 10.20 vs. 19.91 ± 15.08; *p* < 0.05). Occasional alcohol consumption was linked to slightly lower lentigo counts compared to non-drinkers.

Certain clinical factors were associated with an increased number of lentigines. Postmenopausal women (20.44 ± 15.65) and individuals with osteoporosis (20.63 ± 11.03) had higher lentigo counts (Table 11).

Regarding prior treatments, patients who had used NSAIDs had significantly higher lentigo counts (20.12 ± 14.41; *p* < 0.05). Increased lentigo numbers were also noted in patients treated with TNF-α inhibitors, chemoprophylaxis, and PUVA therapy, while those who had received methotrexate or UVB therapy showed lower counts (Table 12).

Patients with biological inflammatory syndrome, cardiovascular disease, renal impairment, and hypertension also exhibited significantly more lentigines (*p* < 0.05). Additionally, those with a history of keratoacanthoma had the highest lentigo counts (34.00 ± 17.34) (Table 13).

## 4. Discussion

The pathogenesis of angiomas remains largely unclear, but they are thought to arise from a combination of genetic predisposition, environmental factors, and systemic health conditions. The prevalence of angiomas increases with age, and they are more commonly observed in individuals with certain comorbidities. Nazer et al. reported that cherry angiomas were more common in patients with a family history of skin lesions and other underlying dermatological conditions, suggesting a possible genetic or environmental influence on their development [21].

Furthermore, some studies have proposed that individuals with more than 50 cherry angiomas may have an increased risk of developing secondary cutaneous malignancies, including melanoma. This observation raises the possibility of a broader dermatological significance of angiomas, suggesting that their presence in high numbers could serve as a potential marker for underlying skin cancer risk and warrant further clinical investigation [22].

Our data showed that women had significantly higher angioma counts compared to men (11.90 ± 6.18 vs. 3.33 ± 2.12; *p* < 0.05), reinforcing previous findings that indicate a greater prevalence of angiomas in female patients. This trend is particularly pronounced in postmenopausal women, suggesting a possible role of hormonal fluctuations in vascular proliferation and skin physiology. Estrogen plays a key role in regulating vascular function, endothelial homeostasis, and angiogenesis, and its decline after menopause has been linked to vascular remodeling and pro-inflammatory cytokine activity. Recent research highlights how estrogen deficiency contributes to increased vascular permeability, inflammation, and impaired microvascular integrity, which may explain the observed gender differences in angioma prevalence [23]. However, as this was a retrospective study, direct hormone level assessments were not available, limiting our ability to establish a direct causal relationship. Despite this limitation, our findings align with the existing literature, which suggests that estrogen depletion may facilitate vascular proliferation [24].

Urban environments are often linked to higher levels of inflammation and skin conditions, which could exacerbate the formation of vascular lesions. A notable contributor to this phenomenon is exposure to air pollutants, which have been demonstrated to further promote angiogenesis and vascular remodeling. Chronic exposure to these environmental stressors can lead to the activation of inflammatory pathways, which may, in turn, promote angiogenesis, a critical factor in the development of angiomas [25].

Lifestyle factors prevalent in urban settings, such as poor dietary habits, reduced physical activity, and a higher incidence of metabolic disorders, could also contribute to vascular alterations that may facilitate excessive angioma development. For instance, diets high in processed foods can lead to increased oxidative stress and inflammation, while sedentary lifestyles are associated with a range of cardiovascular issues that may promote vascular changes [26]. This suggests that environmental factors linked to urban living, such as increased exposure to pollutants and lifestyle stressors, may contribute to the development of angiomas.

Interestingly, smokers had fewer angiomas compared to non-smokers (4.04 ± 3.60 vs. 7.82 ± 6.39; *p* < 0.05), and frequent alcohol drinkers also exhibited lower counts than non-drinkers (4.25 ± 4.01 vs. 9.08 ± 6.54; *p* < 0.05). While smoking and alcohol consumption are generally linked to impaired skin health and increased oxidative stress, these findings are counterintuitive, and their underlying cause remains unclear. Given the retrospective nature of this study, we could not account for potential confounders such as genetic predisposition, dietary factors, or medication use, which may have influenced the results. Future studies with controlled variables are needed to clarify this association.

However, it is possible that the potential immunosuppressive effects of these lifestyle factors alter the inflammatory response in a way that paradoxically reduces angioma formation, possibly by modulating cytokine activity or dampening vascular endothelial growth factor (VEGF) expression [27,28]. Additionally, nicotine has been reported to modulate immune responses, with potential anti-inflammatory effects in various contexts. Moreover, it is essential to reconsider that smoking remains a well-established risk factor for numerous skin conditions, including impaired wound healing and cutaneous malignancies. Therefore, this hypothesis should be interpreted with caution, as further research is needed to elucidate the underlying mechanisms [29]. While smoking and alcohol consumption are generally associated with negative outcomes for skin health, including the exacerbation of conditions like psoriasis, which is linked to increased angiogenesis, the specific relationship between these lifestyle factors and angioma formation requires further investigation [30].

Our results indicate that biologically naïve patients and those receiving TNF-α inhibitors had lower angioma counts compared to those treated with IL-23 inhibitors (5.89 ± 5.53 and 5.06 ± 4.39 vs. 6.94 ± 7.00). This suggests that the type of biological therapy may significantly influence the development of angiomas, potentially due to differences in how these therapies modulate the immune response and inflammatory pathways associated with psoriasis [31]. Biologically naïve patients, defined as those who have not been exposed to biologic therapies, may present with a baseline immune profile that is less prone to chronic inflammation, resulting in lower angioma counts.

IL-23 inhibitors, which are known to have potent effects on the immune system and inflammatory pathways, may promote angiogenesis through the modulation of cytokine profiles, particularly vascular endothelial growth factor (VEGF). Interleukin-23 (IL-23) plays a crucial role in angiogenesis by promoting vascular endothelial growth factor (VEGF) expression through Th17 cell activation. Th17 cells secrete IL-17, a cytokine that stimulates VEGF production, leading to increased vascular proliferation. In psoriasis, where IL-23 is overexpressed, VEGF levels are significantly elevated, contributing to excessive angiogenesis and vascular lesion formation. The interaction between these cytokines and their pro-angiogenic activity strengthens the link between systemic inflammation and vascular changes in psoriatic disease [32,33,34,35].

Inhibition of IL-23 may, therefore, reduce VEGF-driven angiogenesis, potentially limiting the development of angiomas, which are characterized by abnormal blood vessels [34]. IL-23 inhibitors, such as guselkumab, risankizumab, and tildrakizumab, have shown efficacy in treating psoriasis, a condition often associated with elevated VEGF levels and increased angiogenesis [36]. However, further research is necessary to evaluate the direct impact of these inhibitors on angioma formation and the underlying mechanisms involved, including the modulation of inflammatory cytokines and transcription factors, which are known to regulate both IL-23 and VEGF expression [37].

Understanding these interactions could lead to new treatment approaches that address both IL-23 and VEGF pathways, potentially improving conditions associated with excessive angiogenesis and vascular abnormalities. Melly et al. discussed how controlled expression of VEGF can lead to significant vascular proliferation, which may contribute to the formation of angiomas [38]. In patients with psoriasis, the inflammatory environment characterized by elevated levels of pro-inflammatory cytokines, including TNF-α and IL-17, may further enhance VEGF expression, thereby promoting angiogenesis and the development of cherry angiomas [36]. Moreover, the presence of angiomas in patients with psoriasis may be attributed to the underlying mechanisms of angiogenesis that are often activated in inflammatory skin diseases. Psoriasis is characterized by excessive angiogenesis, which contributes to the erythema and scaling seen in psoriatic plaques. The role of angiogenesis in psoriasis is further supported by evidence that suggests increased vascularization in psoriatic skin, which can lead to the development of angiomas [39].

Furthermore, the findings of Das et al. suggest that increased levels of angiogenic factors in the body could trigger the development of angiomas, particularly in patients with underlying vascular conditions. This aligns with the observation that patients with comorbidities such as cardiovascular disease and endocrine dysfunction exhibit higher angioma counts, indicating a systemic influence on angiogenesis [40]. As previously mentioned, biological therapies can lead to paradoxical skin reactions, including the development of new cutaneous lesions. This suggests that while these therapies are effective in managing psoriasis, they may also inadvertently influence the formation of new lesions, including vascular lesions [41].

The finding that women had significantly higher angioma counts compared to men (11.90 ± 6.18 vs. 3.33 ± 2.12; *p* < 0.05) aligns with existing data from the literature that suggest a gender disparity in the prevalence of cherry angiomas. Studies have shown that angiomas are more common in females, particularly in older age groups, which may be attributed to hormonal changes or differences in skin parameters [15].

Our observation that patients diagnosed with psoriatic arthropathy exhibit significantly higher angioma counts compared to those without joint involvement is indicative of the systemic inflammation associated with psoriatic diseases. The reported angioma counts (9.64 ± 6.67 vs. 3.71 ± 3.09; *p* < 0.001) suggest that the inflammatory burden in psoriatic arthritis (PsA) may enhance angiogenic processes, leading to increased vascular lesions such as cherry angiomas. This phenomenon can be explained through several interconnected mechanisms involving cytokine production, immune system activation, and the resultant effects on vascular endothelial growth factor (VEGF) expression [42,43].

Several studies provide evidence supporting this connection. For instance, Shahidi-Dadras et al. demonstrated that serum levels of vascular endothelial growth factor (VEGF) were significantly elevated in psoriatic patients, indicating a potential link between inflammation and angiogenesis [44]. Additionally, Batycka-Baran et al. reported reduced circulating endothelial progenitor cells in patients with plaque psoriasis, further suggesting a disruption in vascular homeostasis associated with the disease [45].

Other research has demonstrated that TNF-α, a key mediator of inflammation in psoriasis, plays a significant role in angiogenesis by promoting the expression of pro-angiogenic factors such as VEGF, contributing to the pathogenesis of angiogenesis in psoriatic skin [46]. Additionally, VEGF overexpression in psoriatic lesions has been linked to disease severity, further supporting the notion that chronic inflammation in psoriatic disease could up-regulate angiogenesis [47]. These findings highlight the importance of further research into how inflammation, vascular remodeling, and angioma formation are interconnected in psoriatic disease. The systemic inflammation associated with joint involvement in psoriatic arthritis (PsA) likely contributes to an environment favoring angiogenesis, resulting in higher angioma counts. Studies have shown that patients with more severe forms of PsA, particularly those with axial and peripheral joint involvement, tend to have a greater inflammatory burden, which correlates with increased vascular lesions. While a study from the literature focused on the prevalence and risk factors associated with PsA, it also noted that clinical phenotypes can vary, with more severe joint involvement potentially leading to increased inflammatory markers, which may reflect greater systemic immune activation and disease progression [48].

Additionally, Mohta et al. discussed the association between psoriasis and cardiovascular ischemia, emphasizing that the same cytokines driving inflammation in psoriasis, such as TNF-α, are also linked to angiogenesis. This reinforces the idea that systemic inflammation in PsA not only affects joint health but also contributes to vascular changes, including increased angioma counts [49,50].

A case report by Flicinski et al. describes a 35-year-old male patient with psoriasis who later developed psoriatic arthritis and underwent treatment with methotrexate and cyclosporine. During therapy, the patient developed multiple vascular nodules on the extremities, which histopathological analysis confirmed as hemangiomas. Notably, these lesions progressed over a three-year period, suggesting a potential link between immunosuppressive therapy and vascular proliferation. Upon discontinuation of cyclosporine, the hemangiomas regressed completely within a few months, leaving behind small depigmented areas. This case highlights the potential angiogenic effects of cyclosporine in psoriatic patients and raises important considerations regarding vascular changes in individuals on long-term immunosuppressive therapy [51]. The vascular proliferation observed in this case highlights the potential for immunosuppressive agents like cyclosporine to promote endothelial hyperplasia, contributing to the formation of vascular lesions. While biologics target specific inflammatory pathways, the parallels in angioma development may reflect a broader systemic effect of immune modulation on vascular health. This observation is significant as it suggests that the immunosuppressive properties of systemic agents may play a role in the development of vascular lesions. The regression of angiomas following the cessation of cyclosporine therapy suggests a direct association between the drug and vascular proliferation. This aligns with the idea that the inflammatory environment in psoriasis, combined with the immunosuppressive effects of other systemic therapies, including methotrexate and biological therapies, may promote angiogenesis and the development of multiple angiomas [52,53].

Patients with a history of PUVA therapy exhibited significantly higher angioma counts (11.86 ± 7.77 vs. 5.43 ± 4.93; *p* < 0.01). PUVA, which combines psoralen and ultraviolet A light, is known to have various effects on the skin, including potential alterations in vascular structures. The increased angioma counts in this group may reflect the long-term effects of phototherapy on skin vasculature [53].

In contrast, those treated with methotrexate or UVB therapy showed lower counts, indicating that certain treatments may have protective effects against the development of cherry angiomas. Research by Banerjee et al. suggests a new hypothesis that patients with a history of PUVA therapy may experience a higher incidence of vascular lesions, including angiomas, due to the cumulative effects of UV exposure and the inflammatory response in the skin [54].

Methotrexate is a widely used systemic therapy for psoriasis, often serving as a first-line treatment before initiating biologic therapy due to its well-established immunosuppressive effects. However, its impact on angioma development remains complex and not entirely understood. It has been proposed that methotrexate may have a protective effect against vascular lesion formation by modulating inflammatory pathways and reducing chronic skin inflammation. This is supported by studies, including those by Gupta et al., which indicate that the reduction in psoriasis severity achieved with methotrexate may indirectly suppress angiogenesis by modulating the inflammatory response [55].

Our cohort findings align with this, as patients treated with methotrexate or UVB therapy had lower angioma counts, suggesting a potential suppressive effect on angiogenesis. This may be due to the downregulation of pro-inflammatory cytokines, particularly TNF-α and IL-6, which are key drivers of angiogenic processes. Additionally, methotrexate’s ability to limit endothelial activation may further reduce vascular lesion formation [55]. Recent research indicates that methotrexate plays a crucial role in restoring the immunosuppressive function of regulatory T cells while preventing excessive proliferation of effector T cells, thereby reducing systemic inflammation and potentially inhibiting angiogenesis [56]. While methotrexate’s potential role in vascular remodeling is promising, additional studies are needed to clarify its mechanisms and long-term effects.

Furthermore, the presence of comorbidities, such as metabolic syndrome, which is highly prevalent among psoriasis patients, may further complicate the relationship between angioma development and therapeutic response. Metabolic syndrome is characterized by low-grade chronic inflammation, insulin resistance, and endothelial dysfunction, all of which may contribute to angiogenesis and the formation of angiomas. Research indicates that psoriasis patients are at an increased risk of metabolic disorders, raising the possibility that systemic inflammation and vascular changes may play a key role in angioma development [57,58].

The correlation between the number of cherry angiomas in psoriasis patients and their treatment history with PUVA or methotrexate is complex and has not been thoroughly studied. PUVA therapy has been associated with an increased incidence of angiomas, likely due to its cumulative impact on skin vasculature and inflammatory pathways over time. On the other hand, methotrexate’s immunomodulatory properties have been postulated to exert a protective role by attenuating pro-inflammatory cytokines such as TNF-α and IL-6, which are known factors in angiogenesis [59,60]. However, the precise mechanisms underlying these effects remain to be elucidated. Future studies should explore the immunologic pathways involved, the role of metabolic dysfunction, and the potential for specific therapeutic interventions to target angiogenesis in psoriasis patients.

Understanding the mechanisms behind the formation of angiomas in psoriasis patients undergoing biologic therapies remains an important area for future investigation. Long-term studies are needed to evaluate how angioma development correlates with treatment types, disease progression, and patient outcomes. Such research could provide valuable insights into the clinical significance of these lesions and their potential role in guiding dermatologic and systemic management strategies.

### Solar Lentigos

Our results indicate that women and rural patients have slightly higher numbers of solar lentigines, which is consistent with the existing literature highlighting gender predisposition and environmental influences on skin conditions. For example, Byrom et al. note that women are often more affected by solar lentigo due to their skin type and sun exposure habits [61]. The underlying biological mechanisms contributing to the development of solar lentigo in patients undergoing biological therapies are complex and multifactorial. Recent studies have highlighted the role of dermal fibroblasts in regulating pigmentation, with senescent fibroblasts upregulating melanogenesis regulators in response to UV exposure [13]. Furthermore, the interaction between UV radiation and environmental factors likely contributes to the development of acquired lentigines [62].

In addition, the observation that smokers exhibit lower counts of solar lentigo compared to non-smokers is intriguing and may indicate a complex interaction between smoking and skin aging processes. While smoking is known to accelerate skin aging through oxidative stress and impaired collagen synthesis, its direct impact on the development of solar lentigo remains unclear. This aspect was also studied by Sadoghi et al., and their results revealed no significant differences in the prevalence of nevus, atypical nevus, and lentigines between smokers and non-smokers in sun-exposed and non-sun-exposed areas [63]. Furthermore, the marginally lower values observed in our cohort for occasional drinkers compared to non-drinkers may suggest that lifestyle factors, such as alcohol consumption, could also contribute to variations in skin health and pigmentation. This paradoxical finding suggests the need for further investigation into how smoking influences melanocyte activity and pigmentation pathways.

Our findings demonstrate that postmenopausal women and patients with osteoporosis have higher counts of solar lentigines, a result that aligns with established research on hormonal changes and skin dynamics during these stages of life [64,65]. This increased vulnerability to photodamage is likely a contributing factor to the increased pigmentation observed in postmenopausal women. Furthermore, osteoporosis, which frequently coexists with menopause, may further exacerbate systemic inflammation, potentially influencing melanocyte activity and promoting lentigo formation [66]. Postmenopausal women experience changes in skin structure and function, primarily due to the decline in estrogen levels. Estrogen is thought to play a protective role in skin health, including maintaining collagen levels and skin hydration. Estrogen depletion during menopause not only accelerates bone density loss, increasing the risk of osteoporosis, but also has a significant impact on skin integrity. Lower estrogen levels are frequently linked to thinner skin, diminished collagen production, and impaired repair mechanisms, all of which render the cutaneous surface more susceptible to UV-induced damage [67]. The decline in estrogen leads to increased skin fragility and greater susceptibility to photodamage, often resulting in lentigines. These changes reflect the broader impact of hormonal fluctuations on skin aging and pigmentation. Given this heightened vulnerability, proactive measures such as consistent sun protection and regular dermatologic evaluations are essential for mitigating long-term effects and preserving skin health in postmenopausal women [68].

The increased number of lentigos observed in patients with biological inflammatory syndrome, cardiovascular disease, renal impairment, and hypertension strongly supports the hypothesis that systemic inflammation and oxidative stress can contribute to the pathogenesis of solar lentigines. Chronic inflammatory states are known to increase levels of pro-inflammatory cytokines, which can disrupt melanocytic activity and promote melanogenesis. Similarly, oxidative stress, a common feature of these conditions, can generate reactive oxygen species (ROS) that may damage keratinocytes and melanocytes, further exacerbating pigmentation changes [69,70]. The correlation of higher lentigo counts with comorbidities is supported by the work of Imokawa, who discusses how inflammatory cytokines released from dysfunctional keratinocytes can lead to melanocytic hyperplasia, potentially exacerbating conditions like solar lentigo. This is particularly relevant in patients with psoriasis, who express elevated cytokine activity and often have concurrent inflammatory conditions, which could lead to excess proliferation of melanocytes and pigmentation [71].

Research indicates that inflammatory cytokines, such as TNF-α and IL-17, can inhibit melanocyte growth and pigmentation gene expression. For instance, Zhang et al. demonstrated that these cytokines negatively affect melanocyte activity, but this inhibition can be reversed upon their withdrawal, suggesting a dynamic interplay between inflammation and pigmentation [40,72]. The correlation between higher lentigo counts and the administration of TNF-α inhibitors is particularly interesting and noteworthy, as it suggests a potential impact of these therapeutic agents on skin pigmentation. These agents are designed to target chronic inflammatory conditions, but by altering key inflammatory pathways, they may also have an impact on melanocytic activity and disrupt the balance of the pigmentation process, demonstrating that therapeutic interventions aimed at reducing chronic inflammation can have unintended consequences on the skin surface [73].

Case reports from the literature further illustrate the potential impact of biologic therapies on lentigo development. For instance, María et al. described a lentiginous eruption in resolving psoriasis plaques during ixekizumab therapy, attributing it to the rapid modulation of cytokine pathways such as IL-17, which may alter pigmentation signaling. The authors noted that as psoriasis plaques resolved under ixekizumab treatment, there was a notable alteration in the local cytokine environment, which may have contributed to the observed changes [74]. Similarly, Dogan and Atakan reported multiple lentigines arising in resolved psoriatic plaques following infliximab treatment, suggesting that the suppression of pro-inflammatory cytokines like TNF-α may influence melanocyte activity and pigmentation [75]. Guttierez-Gonzalez et al. documented lentigines in resolving psoriatic plaques during ustekinumab therapy, also highlighting the potential role of IL-12/IL-23 axis inhibition in melanocyte stimulation and pigmentation [76]. Khanna et al. provided further support with a case of eruptive lentigines confined to resolving plaques after tildrakizumab treatment, suggesting cytokine dysregulation as a central mechanism [77]. Finally, Palmisano et al. reported lentiginous eruptions following risankizumab treatment, emphasizing the interplay between IL-23 neutralization and melanocyte activation [78]. The collective evidence from these cases highlights the possible association between biologic therapies and post-inflammatory pigmentation changes, suggesting that the modulation of cytokines, particularly TNF-α, IL-17/IL-23, IL-17, and IL-23, may play a role in the development of lentigo. Further research is necessary to elucidate these mechanisms and their implications for long-term patient care.

This study has certain limitations that should be considered. The lack of a control group limits our ability to determine a definitive causal link between biologic therapies and the development of lentigines. A comparative analysis with psoriasis patients not receiving biologics would have provided stronger evidence. However, withholding biologic treatment from eligible patients raises ethical concerns, and identifying a cohort of untreated psoriasis patients would be challenging in a real-world setting, as systemic therapies are the standard of care for moderate to severe cases. To address this, our study focused on within-group comparisons and interpreted the findings in the context of the available literature.

In parallel with dermoscopic observations, we also used in vivo confocal microscopy to assess the efficacy of biologic therapy by monitoring the therapeutic response in real time at well-defined time intervals within psoriatic plaques. Beyond the early detection of inflammatory cell disappearance from the epidermis and dermis—indicating the early anti-inflammatory activity of biologics—the early recovery of bright rims surrounding the dermal papillae (edge papillae) was detected on reflective confocal microscopy by week 5 after treatment initiation. This confocal feature, corresponding to the early reappearance of keratinocyte pigmentation at the dermo-epidermal junction (DEJ), could serve as an early indicator of the normalization of keratinization processes and the reduction in keratinocyte proliferative rates, thereby facilitating the proper incorporation of melanin.

**Figure 1:**
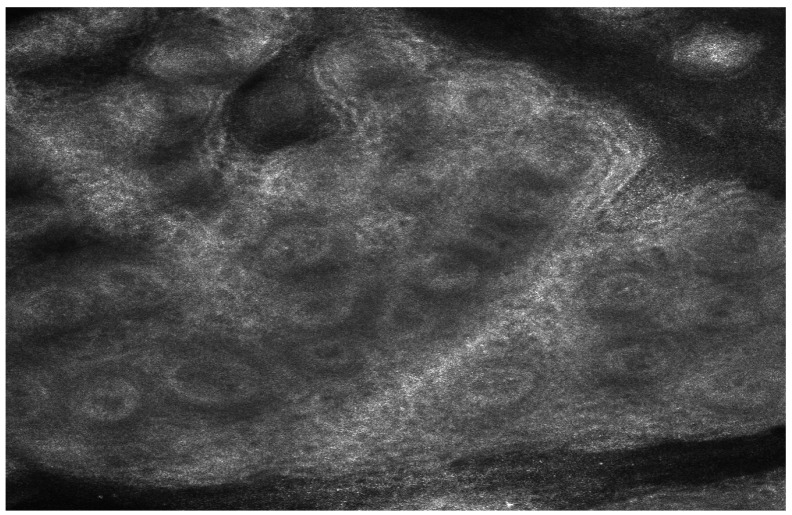
Reflective confocal microscopy aspect before the initiation of biologic therapy—absence of edged papillae visualization, accompanied by pronounced epidermal thickening and inflammatory infiltration.

**Figure 2:**
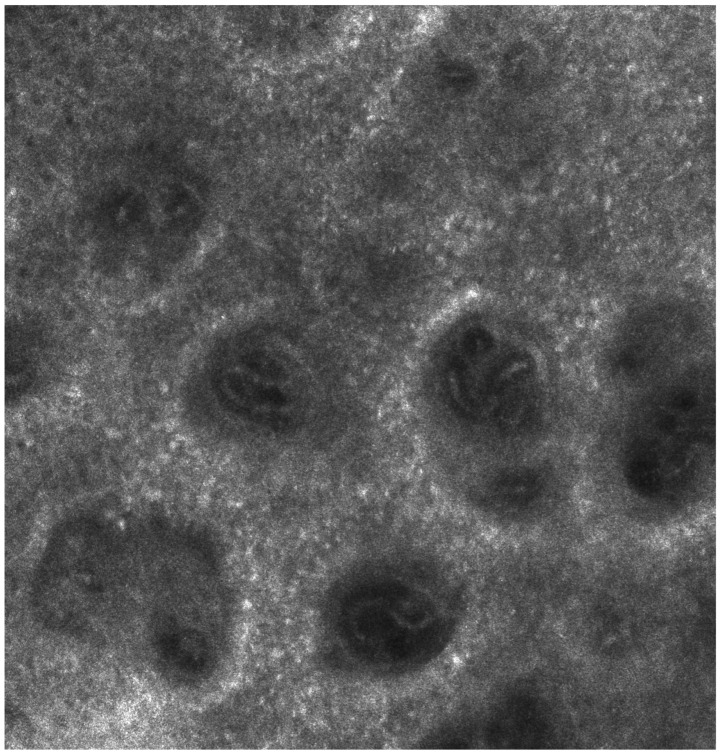
Reflective confocal microscopy aspect before the initiation of biologic therapy—alternative aspect—absence of edged papillae visualization, accompanied by pronounced epidermal thickening and inflammatory infiltration.

In vivo confocal microscopy allowed us to observe structural changes in the epidermis and alterations in melanocyte activity following biologic therapy. These findings suggest a potential link between cytokine modulation and epidermal remodeling. Before starting treatment, the “edged papillae” appearance (bright papillary structures) was absent, likely due to high epidermal turnover and elevated levels of inflammatory cytokines, particularly TNF-α, IL-17, and IL-23 (Figure 1 and Figure 2).

As biologic therapy reduced inflammation and psoriatic plaques began to clear, the “edged papillae” feature became visible again, suggesting a normalization of epidermal renewal. This change indicates that blocking IL-23 and IL-17 helps restore the balance of the epidermal microenvironment, allowing melanocytes to resume interleukin secretion and regain normal function. These findings support the idea that chronic inflammation in psoriasis disrupts not only keratinocyte proliferation but also the dermo-epidermal structure, thereby affecting melanocyte homeostasis [78].

**Figure 3:**
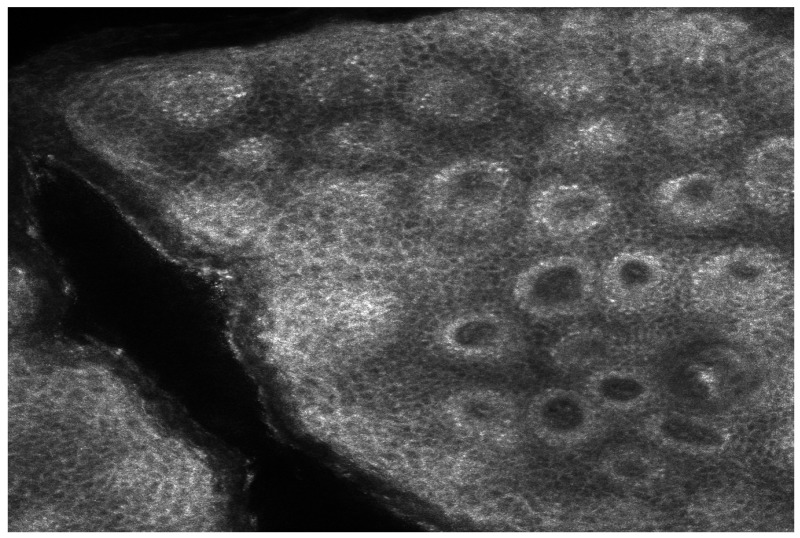
Reflective confocal microscopy aspect of a psoriasis plaque 3 months after treatment initiation—reappearance of the edged papillae structure, alongside a reduction in inflammation and papillomatosis.

**Figure 4:**
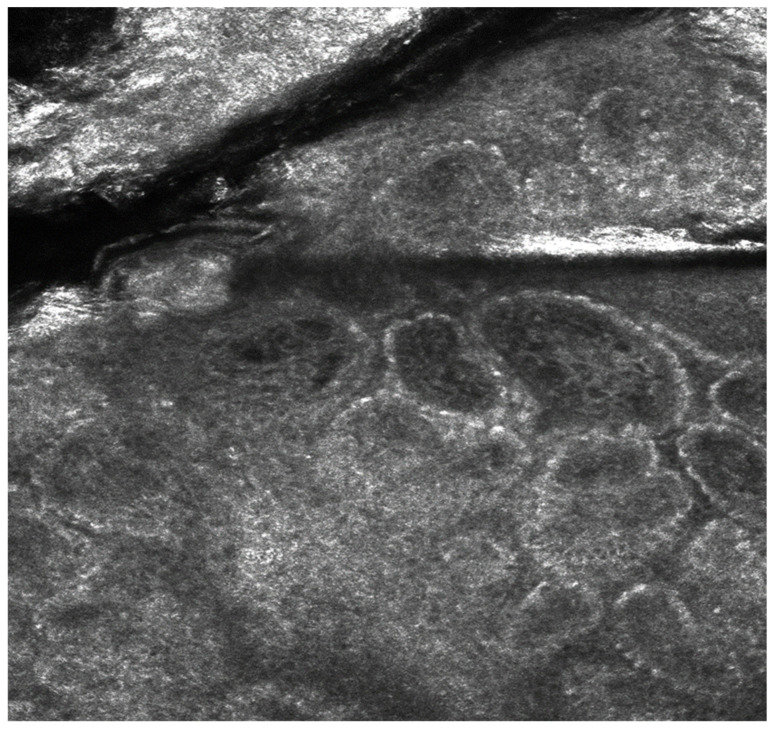
Reflective confocal microscopy aspect of a psoriasis plaque 6 months after treatment initiation—reappearance of the edged papillae structure, alongside a reduction in inflammation and papillomatosis.

These observations highlight the potential of confocal microscopy as a valuable tool for tracking treatment response, offering objective markers of epidermal restoration and melanocyte function. The reappearance of “edged papillae” with biologic therapy also reinforces the idea that epidermal regeneration and melanocyte activity are closely linked to cytokine balance, which may explain post-inflammatory hyperpigmentation and lentiginous changes in psoriasis patients (Figure 3 and Figure 4).

## 5. Conclusions

This study provides new perspectives on the relationship between biologic therapies, systemic inflammation, and the development of angiomas and solar lentigines in psoriasis patients. The significantly higher angioma counts observed in women, particularly postmenopausal individuals and those with osteoporosis, suggest a role for hormonal factors in vascular proliferation. Additionally, patients receiving IL-23 inhibitors exhibited greater angioma counts compared to those on TNF-α inhibitors, indicating a potential pro-angiogenic effect of IL-23 blockade, possibly mediated through VEGF modulation. The increased angioma burden in psoriatic arthritis patients further supports the hypothesis that chronic systemic inflammation contributes to vascular remodeling and angiogenesis. In contrast, methotrexate and UVB therapy were associated with lower angioma counts, suggesting a potential protective effect against vascular proliferation. Similarly, the higher prevalence of solar lentigines in patients with systemic inflammatory conditions, postmenopausal women, and those undergoing TNF-α inhibition highlights the complex relationship between inflammation, oxidative stress, and melanocyte activity. The paradoxical observation that smokers and occasional alcohol consumers exhibited lower lentigo and angioma counts raises important questions regarding the immunomodulatory effects of these lifestyle factors, warranting further investigation. Additionally, in vivo confocal microscopy provided valuable insights into early epidermal remodeling during biologic therapy, with the reappearance of bright rims around dermal papillae emerging as a potential marker of treatment response. These findings highlight the need for further longitudinal studies to clarify the long-term dermatologic effects of biologic therapies, improve patient monitoring, and refine treatment strategies to minimize cutaneous side effects while ensuring optimal disease control.

## Figures and Tables

**Table 1:** Duration of biologic therapy in the study group.

	n	Mean	Standard Deviation	Standard Error of the Mean	Min	Max	Median
Biologic therapy duration (months)	60	62.38	6.01	46.57	10	180	39.50

**Table 2:** Therapeutic classes of biologics in the study group.

		n	%
Therapeutic class	TNF-α inhibitors	18	30.0
	IL-17 inhibitors	25	41.7
	IL-23 inhibitors	17	28.3
Total		60	100.0

**Table 3:** Disease duration in the study group.

	n	Mean	Standard Deviation	Standard Error of the Mean	Min	Max	Median
Disease duration (years)	60	24.62	1.56	12.14	4	46	25.00

**Table 4:** Characteristics of disease severity scores—baseline and during follow-up.

	n	Mean	Standard Deviation	Standard Error of Mean	Min	Max	Median	Wilcoxon Test	
								Z	*p*
**PASI Score**									
baseline	60	26.24	1.36	10.60	7.1	51.3	23.85		
3 months	60	12.70	0.98	7.59	1.3	30.0	12.50	−6.736	<0.001
6 months	60	5.80	0.56	4.38	0.0	18.0	4.50	−6.593	<0.001
12 months	60	2.28	0.30	2.38	0	12	2.00	−5.969	<0.001
**DLQI score**									
baseline	60	21.07	0.80	6.24	1	30	22.00		
3 months	60	9.33	0.88	6.85	0	28	9.00	−6.628	<0.001
6 months	60	4.35	0.57	4.43	0	23	3.00	−5.991	<0.001
12 months	60	2.00	0.38	2.98	0	18	1.00	−5.367	<0.001

**Table 5:** Study of angioma counts based on patients’ demographic characteristics.

Number of Angiomas		N	Mean	Standard Deviation	Standard Error of the Mean	Min	Max	Median	Mann–Whitney Test
Gender	male	40	3.33	2.12	0.33	0	11	3.00	U = 79.000
	female	20	11.90	6.18	1.38	1	24	12.00	*p* < 0.001
Residence	urban	42	6.45	5.97	0.92	0	24	4.00	U = 362.500
	rural	18	5.56	4.91	1.15	1	18	4.00	*p* = 0.801

**Table 6:** Study of angioma counts based on patients’ clinical characteristics.

Number of Angiomas		N	Mean	Standard Deviation	Standard Error of Mean	Min	Max	Median	Mann–Whitney/Kruskal–Wallis Test
Smoking	yes	26	4.04	3.60	0.70	0	19	3.00	U = 297.500
	no	34	7.82	6.39	1.09	0	24	6.00	*p* = 0.030
Alcohol consumption	regularly	36	4.25	4.01	0.66	0	18	3.00	U = 225.000
	does not consume	24	9.08	6.54	1.33	0	24	9.00	*p* = 0.002
Menopause	yes	16	13.44	5.79	1.44	2	24	13.00	U = 6.000
	no	4	5.75	3.40	1.70	1	9	6.50	*p* = 0.011
Osteoporosis	yes	8	10.88	4.79	1.69	4	18	12.00	U = 71.500
	no	52	5.46	5.46	0.75	0	24	4.00	*p* = 0.003
Chronic UV exposure	occasionally	20	5.40	4.72	1.05	1	18	3.50	U = 373.000
	no	40	6.58	6.08	0.96	0	24	4.00	*p* = 0.670
History of sunburns	yes	16	5.31	5.21	1.30	1	18	3.00	U = 304.500
	no	44	6.50	5.82	0.87	0	24	4.00	*p* = 0.424
Histofy of keraoacanthoma	yes	3	5.67	4.61	2.66	3	11	3.00	U = 84.000
	no	57	6.21	5.73	0.76	0	24	4.00	*p* = 0.975
Phototype	II	19	8.26	6.27	1.44	0	24	9.00	H = 3.912
	III	38	5.11	4.70	0.76	1	23	4.00	*p* = 0.141
	IV	3	6.67	10.69	6.17	0	19	1.00	

**Table 7:** Study of angioma counts based on previous medication used and clinical forms.

Number of Angiomas		N	Mean	Standard Deviation	Standard Error of Mean	Min	Max	Median	Mann–Whitney/Kruskal–Wallis Test
Biologic-naïve patient	Yes	47	5.89	5.55	0.81	0	24	4.00	U = 249.000
	No	13	7.23	6.11	1.69	0	19	5.00	*p* = 0.307
Biological therapy class	TNF-α inhibitors	18	5.06	4.39	1.03	2	17	4.00	H = 0.451
	IL-17 inhibitors	25	6.48	5.53	1.10	1	24	5.00	*p* = 0.798
	IL-23 inhibitors	17	6.94	7.00	1.69	0	23	4.00	
History of Beta-blocker use	yes	14	9.93	5.95	1.59	3	23	9.00	U = 135.000
	no	46	5.04	5.09	0.75	0	24	3.00	*p* <0.001
History of NSAID use	yes	25	7.20	5.95	1.19	1	24	5.00	U = 341.000
	no	35	5.46	5.39	0.91	0	23	4.00	*p* = 0.145
Family history of psoriasis	yes	15	7.87	6.95	1.79	0	24	7.00	U = 294.000
	no	45	5.62	5.11	0.76	0	23	4.00	*p* = 0.454
Psoriatric arthropaty	absent	34	3.76	3.12	0.53	0	14	3.00	H = 17.058
	axial	7	11.86	7.94	3.00	3	24	13.00	*p* <0.001
	peripheral	10	10.60	6.27	1.98	2	23	9.00	
	mixed	9	6.00	5.12	1.70	1	17	4.00	
Genital involvement	yes	5	10.80	8.22	3.68	2	24	9.00	U = 78.500
	no	55	5.76	5.26	0.71	0	23	4.00	*p* = 0.117
Scalp involvemenet	yes	50	6.04	5.32	0.75	0	23	4.00	U = 237.500
	no	10	6.90	7.37	2.33	1	24	3.00	*p* = 0.803
Palmoplantar involvement	yes	23	5.52	4.81	1.00	1	19	4.00	U = 412.000
	no	37	6.59	6.14	1.01	0	24	4.00	*p* = 0.836
Nail involvement	yes	50	6.50	5.86	0.82	0	24	4.00	U = 187.500
	no	10	4.60	4.35	1.37	0	12	3.00	*p* = 0.212

**Table 8:** Study of angioma counts based on observed comorbidities.

Number of Angiomas		N	Mean	Standard Deviation	Standard Error of Mean	Min	Max	Median	Mann–Whitney Test
Inflammatory biological syndrome	yes	28	7.00	5.69	1.07	1	24	4.00	U = 360.000
	no	32	5.47	5.60	0.99	0	23	4.00	*p* = 0.189
Endocrine dysfunction	yes	3	17.00	7.21	4.16	9	23	19.00	U = 14.000
	no	57	5.61	5.02	0.66	0	24	4.00	*p* = 0.009
Diabetes	yes	11	6.55	5.14	1.55	2	18	4.00	U = 250.000
	no	49	6.10	5.80	0.83	0	24	4.00	*p* = 0.707
Urinary tract infection	yes	7	6.43	4.82	1.82	1	13	7.00	U = 179.500
	no	53	6.15	5.79	0.79	0	24	4.00	*p* = 0.893
Cardiovascular disease	yes	22	8.68	6.06	1.29	2	23	9.00	U = 231.000
	no	38	4.74	4.91	0.79	0	24	3.00	*p* = 0.004
Locomotory disorders	yes	14	8.21	5.01	1.33	2	18	8.00	U = 195.500
	no	46	5.57	5.74	0.84	0	24	3.50	*p* = 0.026
Asthma	yes	2	13.50	13.43	9.50	4	23	13.50	U = 29.500
	no	58	5.93	5.28	0.69	0	24	4.00	*p* = 0.271
Chronic obstructive pulmonary disease	yes	9	5.67	5.40	1.80	2	19	4.00	U = 228.000
	no	51	6.27	5.74	0.80	0	24	4.00	*p* = 0.975
History of coronary artery disease/stroke	yes	2	6.50	3.53	2.50	4	9	6.50	U = 42.500
	no	58	6.17	5.73	0.75	0	24	4.00	*p* = 0.547
History of surgical procedures	yes	9	7.00	6.00	2.00	2	19	4.00	U = 211.500
	no	51	6.04	5.63	0.79	0	24	4.00	*p* = 0.707
Renal disease	yes	2	10.00	1.41	1.00	9	11	10.00	U = 24.000
	no	58	6.05	5.70	0.74	0	24	4.00	*p* = 0.191
Neurological disorders	yes	1	4.00			4	4	4.00	U = 29.500
	no	59	6.22	5.69	0.74	0	24	4.00	*p* = 1.000
History of hepatic disease	yes	35	6.63	6.16	1.04	1	24	4.00	U = 398.500
	no	25	5.56	4.90	0.98	0	18	4.00	*p* = 0.556
Viral hepatic infections	yes	3	2.00	0.00	0.00	2	2	2.00	U = 31.500
	no	57	6.40	5.71	0.75	0	24	4.00	*p* = 0.066
Anemia	yes	5	8.20	6.53	2.92	2	18	7.00	U = 107.000
	no	55	6.00	5.59	0.75	0	24	4.00	*p* = 0.434
Thrombocytopenia	yes	4	9.25	9.32	4.66	3	23	5.50	U = 80.000
	no	56	5.96	5.36	0.71	0	24	4.00	*p* = 0.365

**Table 9:** Study of angioma counts based on previous therapies.

Number of Angiomas		N	Mean	Standard Deviation	Standard Error of Mean	Min	Max	Median	Mann–Whitney Test
Chemoprophylaxis with isoniazid for tuberculosis prevention	yes	37	7.41	6.18	1.01	2	24	4.00	U = 271.500
	no	23	4.22	4.07	0.85	0	14	3.00	*p* = 0.018
Previous Methotrexate (MTX) Treatment	yes	57	6.18	5.72	0.75	0	24	4.00	U = 77.000
	no	3	6.33	5.13	2.96	2	12	5.00	*p* = 0.798
Previous PUVA Therapy	yes	7	11.86	7.77	2.93	0	24	11.00	U = 88.500
	no	53	5.43	4.93	0.67	0	23	4.00	*p* = 0.023
Previous UVB Therapy	yes	10	5.30	4.76	1.50	0	14	3.50	U = 226.000
	no	50	6.36	5.84	0.82	0	24	4.00	*p* = 0.631

**Table 10:** Study of solar lentigo counts based on patients’ demographic characteristics.

Number of Solar Lentigos		N	Mean	Standard Deviation	Standard Error of Mean	Min	Max	Mean	Mann–Whitney Test
Gender	male	40	15.13	13.21	2.090	0	50	12.00	U = 356.000
	female	20	17.85	15.17	3.393	0	62	12.50	*p* = 0.490
Residence	urban	42	15.74	14.91	2.302	0	62	11.00	U = 327.500
	rural	18	16.72	11.24	2.650	2	43	14.00	*p* = 0.415

**Table 11:** Study of solar lentigo counts based on patients’ clinical characteristics.

Number of Solar Lentigos		N	Mean	Standard Deviation	Standard Error of Mean	Min	Max	Median	Mann–Whitney/Kruskal–Wallis Test
Smoking	yes	26	10.96	10.20	2.00	0	43	8.50	U = 275.000
	no	34	19.91	15.08	2.58	0	62	15.50	*p* = 0.013
Alcohol consumption	occasionally	36	14.33	11.25	1.87	0	43	13.00	U = 393.000
	does not consume	24	18.58	16.93	3.45	0	62	11.50	*p* = 0.556
Menopause	yes	16	20.44	15.65	3.91	6	62	17.00	U = 13.000
	no	4	7.50	7.32	3.66	0	16	7.00	*p* = 0.080
Osteoporosis	yes	8	20.63	11.03	3.90	11	45	20.00	U = 134.000
	no	52	15.33	14.16	1.96	0	62	11.00	*p* = 0.107
Chronic UV exposure	occasionally	20	15.40	11.39	2.54	2	43	13.00	U = 391.000
	no	40	16.35	15.02	2.37	0	62	11.50	*p* = 0.888
History of sunburn	yes	16	15.25	9.90	2.47	2	38	13.00	U = 330.000
	no	44	16.32	15.09	2.27	0	62	11.00	*p* = 0.713
History of keratoacanthoma	yes	3	34.00	17.34	10.01	14	45	43.00	U = 29.000
	no	57	15.09	13.13	1.74	0	62	11.00	*p* = 0.056
Phototype	II	19	15.79	15.25	3.49	0	62	11.00	H = 0.262
	III	38	16.47	13.53	2.19	0	50	13.50	*p* = 0.877
	IV	3	12.00	11.53	6.65	0	23	13.00	

**Table 12:** Study of solar lentigo counts based on previous treatments and clinical characteristics.

Number of Solar Lentigos		N	Mean	Standard Deviation	Standard Error of Mean	Min	Max	Median	Mann–Whitney/Kruskal–Wallis Test
Biologic-Naïve Patient	yes	47	16.38	15.07	2.19	0	62	11.00	U = 279.000
	no	13	14.77	8.22	2.28	0	26	16.00	*p* = 0.634
Biological therapy class	TNF-α inhibitors	18	20.72	13.12	3.09	6	45	17.00	H = 4.744
	IL-17 inhibitors	25	14.16	13.81	2.76	0	62	11.00	*p* = 0.093
	IL-23 inhibitors	17	13.82	14.16	3.43	0	50	11.00	
History of Beta-blocker use	yes	14	21.29	15.45	4.13	2	62	20.00	U = 216.500
	no	46	14.43	13.06	1.92	0	50	11.00	*p* = 0.065
History of NSAID use	yes	25	20.12	14.41	2.88	0	62	17.00	U = 282.500
	no	35	13.11	12.81	2.16	0	50	11.00	*p* = 0.020
Family history of psoriasis	yes	15	15.47	13.96	3.60	0	50	12.00	U = 325.000
	no	45	16.22	13.93	2.07	0	62	13.00	*p* = 0.831
Psoriatic arthropaty	absent	34	15.12	14.67	2.51	0	50	11.00	H = 2.756
	axial	7	19.14	11.24	4.25	0	36	21.00	*p* = 0.431
	peripheral	10	20.10	16.86	5.33	6	62	16.00	
	mixed	9	12.56	7.86	2.62	3	26	11.00	
Genital involvement	yes	5	16.40	14.08	6.29	3	36	11.00	U = 137.000
	no	55	16.00	13.93	1.88	0	62	13.00	*p* = 1.000
Scalp involvement	yes	50	15.66	13.69	1.93	0	62	12.00	U = 228.500
	no	10	17.90	15.11	4.78	0	45	12.50	*p* = 0.669
Palmoplantar involvement	yes	23	15.70	12.06	2.51	2	50	13.00	U = 409.500
	no	37	16.24	14.98	2.46	0	62	11.00	*p* = 0.807
Nail involvement	yes	50	15.32	11.77	1.66	0	50	12.50	U = 241.000
	no	10	19.60	21.95	6.94	0	62	10.50	*p* = 0.858

**Table 13:** Study of solar lentigo counts based on observed comorbidities.

Number of Solar Lentigos		N	Mean	Standard Deviation	Standard Error of Mean	Min	Max	Median	Mann–Whitney Test
Inflammatory biological syndrome	yes	28	20.93	14.25	2.69	0	62	19.00	U = 243.000
	no	32	11.75	12.09	2.13	0	50	8.50	*p* = 0.002
Endocrine dysfunction	yes	3	14.00	8.18	4.72	7	23	12.00	U = 82.500
	no	57	16.14	14.10	1.86	0	62	13.00	*p* = 0.924
Diabetes	yes	11	21.18	17.90	5.39	0	62	16.00	U = 204.500
	no	49	14.88	12.68	1.81	0	50	11.00	*p* = 0.214
Urinary tract infection	yes	7	26.00	21.90	8.27	0	62	22.00	U = 128.000
	no	53	14.72	12.10	1.66	0	50	11.00	*p* = 0.194
Cardiovascular disease	yes	22	21.68	14.80	3.15	2	62	18.00	U = 238.000
	no	38	12.76	12.27	1.99	0	50	9.50	*p* = 0.006
Locomotory disorders	yes	14	21.43	16.22	4.33	2	62	21.00	U = 222.000
	no	46	14.39	12.76	1.88	0	50	11.00	*p* = 0.080
Asthma	yes	2	17.00	14.14	10.00	7	27	17.00	U = 51.000
	no	58	16.00	13.94	1.83	0	62	12.50	*p* = 0.793
Chronic obstructive pulmonary disease	yes	9	21.44	17.74	5.91	6	62	14.00	U = 173.500
	no	51	15.08	13.00	1.82	0	50	11.00	*p* = 0.246
History of coronary artery disease/stroke	yes	2	32.00	42.42	30.00	2	62	32.00	U = 51.500
	no	58	15.48	12.53	1.64	0	50	12.50	*p* = 0.793
Hitory of surgical procedures	yes	9	26.78	16.53	5.51	11	62	23.00	U = 104.000
	no	51	14.14	12.55	1.75	0	50	11.00	*p* = 0.009
Renal disease	yes	2	53.50	12.02	8.50	45	62	53.50	U = 1.500
	no	58	14.74	12.02	1.57	0	50	11.50	*p* = 0.002
Neurological disorders	yes	1	27.00			27	27	27.00	U = 10.000
	no	59	15.85	13.87	1.80	0	62	12.00	*p* = 0.367
History of hepatic disease	yes	35	14.34	12.92	2.18	0	45	11.00	U = 352.000
	no	25	18.40	14.95	2.99	0	62	17.00	*p* = 0.199
Viral hepatic infections	yes	3	17.67	24.00	13.86	0	45	8.00	U = 75.500
	no	57	15.95	13.44	1.78	0	62	13.00	*p* = 0.749
Anemia	yes	5	23.80	12.98	5.80	13	45	21.00	U = 73.500
	no	55	15.33	13.79	1.86	0	62	11.00	*p* = 0.087
Thrombocytopenia	yes	4	11.25	3.09	1.54	7	14	12.00	U = 100.500
	no	56	16.38	14.24	1.90	0	62	12.50	*p* = 0.742

## Data Availability

No new data were created or analyzed in this study. Data sharing is not applicable to this article.

## Abbreviations

The following abbreviations are used in this manuscript:

ROS	Reactive oxygen species
UV	Ultraviolet radiation
PUVA	Psoralen plus ultraviolet A therapy
UVB	Ultraviolet B therapy
PsA	Psoriatic arthritis
MTX	Methotrexate
PASI	Psoriasis Area and Severity Index
DLQI	Dermatology Life Quality Index
TNF	Tumor necrosis factor
VEGF	Vascular endothelial growth factor
IL	Interleukin
Th	T helper cells
